# Quantifying the Effects of Medical Examination and Possible Risk Factors against the Incidence of Cervical Cancer in a Low Human Papillomavirus Vaccination Coverage: An Ecological Study in Japan

**DOI:** 10.3390/cancers13194784

**Published:** 2021-09-24

**Authors:** Yueming Yu, Ryota Matsuyama, Miwako Tsunematsu, Masayuki Kakehashi

**Affiliations:** Department of Health Informatics, Graduate School of Biomedical and Health Sciences, Hiroshima University, Hiroshima 734-8553, Japan; d191393@hiroshima-u.ac.jp (Y.Y.); tsunematsu@hiroshima-u.ac.jp (M.T.); kakehashi@hiroshima-u.ac.jp (M.K.)

**Keywords:** cervical cancer, screening test, sexually transmitted diseases, ecological study, low HPV vaccine coverage

## Abstract

**Simple Summary:**

Cervical cancer (CC) is one of the most common gynecological malignancies in females, mainly caused by human papillomavirus (HPV). In countries with lower HPV vaccine coverage, such as Japan, medical examination may play a key role in decreasing CC incidence. The aim of this study is to measure the effect of medical examination (i.e., screening and detailed examination) on CC incidence in Japan by considering the effects of risk factors for the development of CC. We clarified associations between CC and possible risk factors by Pearson’s correlation coefficients and generalized linear models. Taking the time-dependent effects of Chlamydia, Gonorrhea, Condyloma, population economic status, and smoking rate into account, the effect of screening testing on CC incidence was estimated. The increase in screening rate was considered to decrease CC incidence effectively but not drastically, suggesting the need for the combined use of other efficient preventive measures such as HPV vaccination.

**Abstract:**

Cervical cancer (CC) is one of the most common gynecological malignancies in females, mainly caused by human papillomavirus (HPV). In countries with lower HPV vaccine coverage, such as Japan, medical examination may play a key role in decreasing CC incidence. This study aimed to quantify the effect of medical examination on cervical cancer (CC) incidence in Japan, considering the effects of possible risk factors. By collecting Japan’s Prefectural data on CC incidence (2013–2017), incidence of sexually transmitted diseases (STDs; Chlamydia, Herpes, Condyloma, and Gonorrhea; 1993–2012), screening and detailed examination rate against CC (2013–2016), smoking rate (2001–2013), economic status (disposable income and economic surplus; 2014–2015), and education status (2015), we analyzed associations among them using Pearson’s correlation coefficients. Additionally, assuming that the incidence of STDs reflects the frequency of risky sexual behavior at the co-infection point with HPV, we constructed generalized linear models to predict CC incidence, taking a 5–20-year time-lag between incidences of STDs and the CC incidence. Against CC incidence, Chlamydia in females and Gonorrhea in males with a 15-year time-lag showed positive associations, while Condyloma in both genders with a 15-year time-lag, screening rate, economic status, and smoking rate showed negative associations. An increase in screening test rate by 10% was estimated to decrease CC incidence by 9.6%. This means that screening tests decrease CC incidence effectively, but not drastically, suggesting the need for additional countermeasures for CC prevention.

## 1. Introduction

Human papillomavirus (HPV) is a double-stranded DNA virus belonging to the Papillomaviridae family, and the primary causal factor for the development of cervical cancer (CC) [1]. It has been reported that approximately 20–40% of cervical precancerous lesions (i.e., cervical intraepithelial neoplasia) [2,3] and 90% to 98% of CC are associated with HPV infection [4]. Since the main route of HPV transmission is considered to be sexual contact, HPV infection is regarded as a sexually transmitted disease (STD) [4]. HPVs consist of a large number of varieties, and not all the virus types are associated with the development of CC: there are more than one hundred known genotypes of HPV, classified as “high-risk (HR)” and “low-risk (LR)” varieties based on the frequency of their identification in CCs [5]. The LR-types are associated with benign hyperplastic growths such as genital warts (i.e., Condyloma acuminatum), while the HR-HPV may cause cervical intraepithelial neoplasia (CIN) and can result in the development of CC [6].

To prevent the infection of HR-HPVs and subsequent development of CC, the successful development of HPV vaccines has led to a breakthrough in the prevention of CC [7]. Unlike other cancers, CC can be prevented by early use of the HPV vaccine and may potentially be eradicated entirely [8]. Since the U.S. Food and Drug Administration approved the first HPV vaccine for four types of HPV in 2006 [9], public health organizations in many countries and the World Health Organization (WHO) have been urging the use of HPV vaccines and raising awareness of the advantage of vaccination. To date, three types of vaccine have been established: the bivalent, the quadrivalent, and the novevalent vaccines. The bivalent vaccine prevents infection with HPV types 16 and 18, the two HR HPVs that cause approximately 70% of CC [10]. In addition to those HPV types, the quadrivalent vaccine protects against HPV types 6 and 11 [11], and the novevalent one protects against HPV types 6, 11, 31, 33, 45, 52, and 58 [12].

In spite of their proven efficacy, some countries have not yet implemented an HPV vaccine strategy. For instance, the vaccination coverage in Japan is less than 1% for females born after 2002 (as of 20 June 2021) [13]. The bivalent, the quadrivalent, and the novevalent HPV vaccines were licensed in Japan in 2009, 2011, and 2020, respectively [14,15,16]. In 2013, both HPV vaccines were added to the national list of routine vaccination programs by the Ministry of Health, Labor, and Welfare (MHLW) and provided to girls aged 12–16 years 2with financial support [17]. However, the occurrence of so-called “serious adverse events” following HPV vaccination was reported and widely announced by mass-media, stoking public doubts about the safety of the vaccines [18]. In June 2013, MHLW suspended the proactive recommendation of HPV vaccination [19]. As a result of this determination, the inoculation rate of HPV vaccines has decreased sharply [20]. 

Given this lack of HPV vaccination in the population, possible alternative ways to decrease CC incidence will be (i) to decrease the exposure to the possible risk factors of CC, and (ii) to increase the rate of medical examination (e.g., the screening test rate and subsequent detailed examination rate). As for (i), co-infection between HPV and STDs, such as Chlamydia infection and Herpes-simplex virus infection, may occur along with the infection of HPV through risky sexual behavior and may also increase the persistence of HPVs in the cervix [21,22,23,24]. Furthermore, smoking [25,26], economic status [27,28], and education level [29] have been considered to be factors closely linked to the incidence of CC. As for (ii), in Japan, a Pap Smear test and Culpascope test (or HPV test, these days) have been conducted as a screening test and detailed examination test of CC [30]. Increasing the screening test rate and detailed examination rate may be effective interventions to detect precancerous conditions and CC [31]. 

Although both (i) and (ii) are considered to be effective, there is no clear evidence for the effectiveness of these strategies. Considering the current severe burden of CC worldwide and the lack of vaccination programs in some countries, clarifying the association between CC incidence, possible risk factors, and medical examination rate is an imperative task. Therefore, in the present study, we aimed to elucidate how STD incidence, smoking rate, CC screening rate, detailed examination rate, income, and education level are associated with the incidence rate of CC, quantifying the effect of medical examination rate (i.e., screening test rate and detailed examination rate) toward the decrease of CC incidence in Japan. By collecting the data on the possible risk factors at Japan’s prefectural population level, we conducted an ecological study by exploring the factors that might explain any difference found in CC incidence among prefectures in Japan.

## 2. Materials and Methods

As mentioned above, we hypothesized that the following risk factors will influence the incidence of CC: (i) An incidence of a STD in a population will reflect the incidence of risky sexual behavior in the population, which may influence on the infection of HPV in the population. Additionally, the incidences of a STD during a certain period (e.g., multiple years) will demonstrate the persistence of the STD in the population in that duration, which may have a positive impact on the persistence of HPV and the development of CC in the population [32]. (ii) Smoking rate in a population demonstrates the frequency of smoking (both active and passive), which may be associated with the promotion of CC in the population. (iii) Residents’ economic status may influence their examination and treatment behavior for CC. (iv) Examination rates (i.e., both the rate of taking a screening test and that of undertaking a detailed examination) will have a positive influence on the detection of precancerous conditions and CC [30]. (v) Populations with higher education levels will avoid partaking in risky sexual behavior and will tend to undertake medical examinations, which will negatively influence on the HPV infection and persistence. Taking these hypotheses into account, we collected the yearly data of CC incidence and those related to the variables listed in (i) to (v) at the Japanese prefectural level. Subsequently, we examined the associations among them by checking the bivariate associations by Pearson correlation coefficients. Finally, we developed generalized linear models (GLMs) to predict the incidence of cervical cancer by setting the female population in each prefecture as offset. This modeling-based analysis was performed to quantify the effect of medical examination rates on the CC incidence by adjusting the influence of other risk factors (i.e., the influence of risk factors except for medical examination rates can be considered as possible confounders). Using the constructed models, we predicted the effect of medical examination rates on decreasing CC incidence. 

### 2.1. Data Collection

Data used for the present study are listed in Table 1. We collected the prefectural-level CC incidence data between 2013 and 2017 from the Cancer Registry and Statistics of Cancer Information Service provided by the National Cancer Center of Japan [33,34]. As for the STDs included in the present study, we targeted four types of diseases: Condyloma acuminatum, Chlamydia infection, Gonorrhea infection, and Herpes-simplex infection (hereinafter, we refer to them as “Condyloma”, “Chlamydia”, “Gonorrhea”, and “Herpes”, respectively). We focused on these STDs because the diseases usually develop acute symptoms and the incidence of these STDs likely reflects the possible risky sexual behaviors (i.e., only a short time-lag exists between the development of the initial symptoms and the sexual contact). We also found that data on syphilis are available, however, because the disease has a long latent period, the data were not included in the present study. Considering that the infection of HR-HPV occurs with risky sexual behavior, and then persistence of HPV takes 5–20 years to develop to CC [32], the infection of the STDs as an index of risky sexual behavior and as a factor for the persistence of HPV might have occurred between 1993 and 2012. We collected the yearly prefectural-level incidence of each STD from 1993 to 2012 from the data summarized in the National Epidemiological Surveillance of Infectious Diseases (NESID) Program [35,36].

As for the medical examination for CC, we collected the data on the cancer screening rate and detailed examination rate from the Comprehensive Survey of Living Conditions survey [37]. The screening test rate and detailed examination rate for CC were examined once every three years. We focused on the prefectural-level CC screening rate and detailed examination rate in 2013 and 2016.

To determine the relationship between smoking (both direct and secondhand) and the CC incidence among Japanese women, data on smoking in both males and females were used in the present study. The survey for the proportion of smokers in the prefectural population and their smoking behavior have been reported every three years since 2001 in official statistics in Japan (the Comprehensive Survey of Living Conditions [37]). The proportion of smokers who answered as “smoke every day/sometimes” in the investigation were extracted and the averaged values between 2001 and 2013 were used in the following analyses: we assume that smoking will be a promoting factor of CC and the averaged proportion of smokers will represent the long-term smoking behavior of each prefectural population.

Socio-demographic factors were also considered in the present study for their possible influence on CC incidence. As for the economic status, we included two types of data: (i) disposable income in 2015 from the Statistics Bureau of Japan [38] and (ii) average economic surplus defined by the difference between disposable income and the average expenditure in 2014 [39]. Regarding the education level of each prefecture, we hypothesized that the people whose education level is high school graduate or less (hereinafter, refers to equal to or under high school graduate) may have fewer opportunities to acquire knowledge on the prevention of CC compared to the people who received higher education. The proportion equal to or under high school graduate was calculated from the education-level data cited in the Population Census of Japan [40].

Lastly, we collected the data on the equal to or over 25 years old female population of each prefecture from the Statistics Bureau of Japan [41]. This is because (i) the prefectural-level incidence of CC reflects the population size of each prefecture, and (ii) the proportion of CC cases whose age was less than 25 years old at the diagnosis is under 2.5% in Japan. The correction of the incidence by population size (i.e., incidence rate) was required for the comparison of incidence among prefectures.

### 2.2. Statistical Analysis

We observed the existence of missing data in STDs (1.81%). If we remove the prefectural data that contained one or more missing values (i.e., listwise deletion), only 19 out of 47 prefectures remained and showed a large loss of information. To address this problem, those missing data were imputed by the Random Forest algorithm implemented in the “missForest” package [42] installed in R version 4.0.3 [43]. For further analysis, we included data of all the 47 prefectures obtained by the imputation.

Associations among the possible risk factors and the incidence of CC were evaluated by two approaches: the correlation analysis and the multivariate generalized linear modeling (GLM). Firstly, we evaluated the associations between variables by calculating Pearson’s correlation coefficients (*r*). Secondly, we performed Poisson regression to explain the CC incidence between 2013 and 2017 as a response variable and other possible risk factors (i.e., incidence of STDs, screening test rate, detailed examination rate, smoking rate, economic status (either disposable income or average economic surplus), and education level) as explanatory variables. We set t population size of each prefecture as an offset because the CC incidence was influenced by the population of ≥25 years old females in each prefecture. When we observed the existence of overdispersions in the estimated result, we employed a negative binomial error instead of using a Poisson error. We included a possible risk factor as an explanatory variable if the factor and the cervical cancer incidence in any year between 2013 and 2017 showed an association with the absolute value of Pearson’s *r* over 0.10 (i.e., |*r*| > 0.10). That means that any possible risk factor that showed only a correlation equal to or less than 0.10 with CC incidence was not included in the prediction of CC incidence.

In the GLM, we separately designed models according to our research hypothesis based on four types of STD data. In the models, observed prefectural-level CC incidences (in female) were explained as follows: (i) incidence rate of each STD in the population (i.e., the summation of STD incidences in female and those in male), (ii) incidence rate of STD in female only, (iii) incidence rate of STD in male only, and (iv) incidence rates of STD in both sexes (i.e., using incidence in female and that in male independently). In addition, as explained above, we examined the effect of time-lag between the year in which STD incidences were reported and the year in which CC incidence was reported, based on our hypothesis “an incidence of STD in a population may reflect the incidence of risky sexual behavior in the population, which may influence on the infection of HPV in the population and the development of CC in the future.” We considered the time-lags from 5 years to 20 years that corresponded to the duration of CC development. For instance, when we hypothesized the 15-year time-lag for the CC incidences between 2013 and 2017, we used the STD incidence data between 1998 and 2002 as explanatory variables by maintaining the time-lag (i.e., STD in 1998 vs. CC in 2013, STD in 1999 vs. CC in 2014, STD in 2000 vs. CC in 2015, STD in 2001 vs. CC in 2016, and STD in 2002 vs. CC in 2017). As a result, we obtained 16 time-lag patterns for each STD data type.

In addition, considering the possible variance of CC development, we not only used the use of single-year STD incidence data, we also used the moving average of the STD incidences from 2 to 16 years for the analyses. This was based on the other above-mentioned hypothesis, “the incidences of a STD during a certain period (e.g., multiple years) will demonstrate the persistence of the STD in the population in that duration, which may have a positive impact on the persistence of HPV and the development of CC in the population.” For integrating the moving-averaged STD incidences in the model, we considered the time-lag for all multiple-year STD incidence data, taking at least a 5-year and at most a 20-year time-lag between the CC incidence and STD incidences (i.e., at least 5 years and at most 20 years were secured as the duration of CC development). For example, when we used the average of 15-year STD incidences, we considered 2 patterns of time-lags: 5-year time-lag (i.e., STD between 1994 and 2008 vs. CC in 2013, STD between 1995 and 2009 vs. CC in 2014, STD between 1996 and 2010 vs. CC in 2015, STD between 1997 and 2011 vs. CC in 2016, and STD between 1998 and 2012 vs. CC in 2017) and six-year time-lag (i.e., STD between 1993 and 2007 vs. CC in 2013, STD between 1994 and 2008 vs. CC in 2014, STD between 1995 and 2009 vs. CC in 2015, STD between 1996 and 2010 vs. CC in 2016, and STD between 1997 and 2011 vs. CC in 2017). The reason why we used the moving-average of multiple-year incidences was that the averaging can avoid the problem of multi-collinearity in the regression analyses that can be caused by highly correlated STD incidences in close years. The moving average also maintained the scale of any patterns of multiple-year STD incidences, which was convenient for the statistical analyses.

As a result, we considered 136 chronological patterns of STD data (See Supplementary Table S1) in the analysis of the present study. Adding to the 16 time-lag patterns regarding the single-year STD data, the multiple-year STD data brought us 120 patterns (i.e., 1 + 2 + 3 + …+ 15 patterns) of STD data. Furthermore, STD data regarding gender had four types, and economic indices had two patterns (i.e., disposable income and economic surplus). Regarding the screening test rate (2013 or 2016), the detailed examination rate (2013 or 2016), the smoking rate (average between 2001 and 2013), the education level (2012), and the prefectural population (2015), we considered only a single pattern. As for the screening test rate and the detailed examination rate, those in 2013 were considered as the explanatory variables for the CC incidence in 2013 and in 2014, while those in 2016 were considered as the explanatory variables for the CC incidence in 2015, in 2016, and in 2017, assuming the medical examination rates have a similar trend between close years. Using these variables, we obtained 1088 datasets (i.e., 136 × 4 × 2 × 1 × 1× 1 × 1 × 1 patterns). 

For each dataset, we initially constructed a Poisson/negative binomial GLM using a log-link function with all variables in the dataset, i.e., (CC incidence in each prefecture between 2013 and 2017) ~ log{$\overset{4}{\underset{n=1}{\sum }}\beta_{1.n}\left(\mathrm{STD}\;\mathrm{data}\;\mathrm{on}\;\mathrm{the}\;\mathrm{disease}\;s_{n}\right)$ + *β*_2_ (screening test rate) + *β*_3_ (detailed examination rate) + *β*_4_ (smoking rate) + *β*_5_ (economic status) + *β*_6_ (education level) + *offset*}, where $s_{1},s_{2},s_{3},s_{4}$, and *offset* denote Condyloma, Chlamydia, Gonorrhea, Herpes, and *log* (female population in each prefecture), respectively. Subsequently, a parsimonious model was selected by the variable selection using the backward stepwise algorithm. We compared the selected models obtained from each dataset and further selected the most parsimonious model as the most predictive model. All the statistical analyses were performed with R software version 4.0.3 [43]. The estimation of coefficients was performed using glm() (Poisson regression) or glm.nb() (negative binomial regression) functions composed in the “MASS” package [44]. Multi-collinearity among explanatory variables was assessed using the variance inflation factor (VIF) obtained by the vif() function in the “rms” package [45]. We defined the lack of multi-collinearity between predictors as a VIF of less than 5. The selection of the parsimonious model was performed using Akaike information criterion (AIC), which is one of the most common criteria to select one or a few good, optimal regression models from a set of candidate models [46]. The backward stepwise selection was performed by the step() function composed in the “MASS” package [44]. The level of statistical significance in our study was set as *p* < 0.05.

## 3. Results

### 3.1. Correlation Analysis

**Figure 1:**
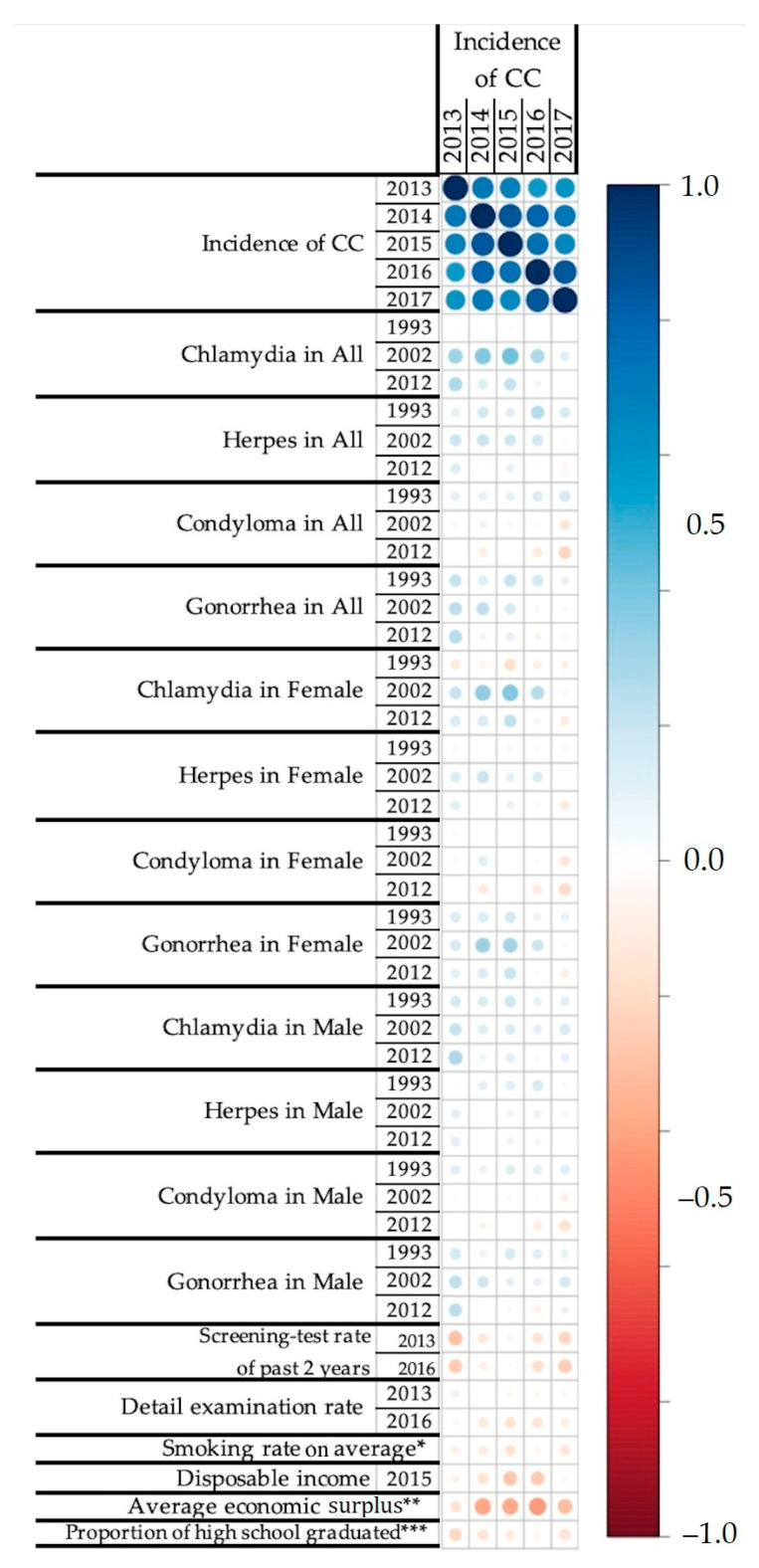
Associations between the incidence rate of CC and the possible risk factors. The blue circle represents a positive correlation between the variables (from 0.0 to 1.0), and the red circle represents a negative correlation between the variables (from –1.0 to 0.0). The darker color shows the stronger correlation. The figure includes the incidence rates of four types of STDs (i.e., Chlamydia, Herpes, Condyloma, and Gonorrhea), two types of economic status (disposable income and average economic surplus), smoking rate, two types of medical examination rates (screening test rate and detailed examination rate), and proportion of the people whose final education level is equal to or under high school graduate. The four digits at the end of each variable name show the year in which the data were reported (e.g., 2013 means the data reported in the year 2013). * The average value of smoking rate among the records in 2001, 2004, 2007, 2010, and 2013. ** The difference between disposable income and average expenditure in 2014. *** Proportion of the people whose final education level is equal to or under high school graduate.

Pearson’s correlations were calculated among the incidence rate of CC, medical examination rate (screening test rate and detailed examination rate), and possible risk factors, as fully shown in Supplementary Table S1 and partially demonstrated in Figure 1. Correlation coefficients between incidence rates of each STD and those of CC showed either positive or negative values. Between the incidence rates of Chlamydia and those of CC, correlation coefficients ranged from −0.21 (Chlamydia in 1999 in female vs. CC in 2017) to 0.42 (Chlamydia in 2003 in all vs. CC in 2015). As for Condyloma incidence rates, correlation coefficients ranged from −0.31 (Condyloma in 1999 in female vs. CC incidence rate in 2017) to 0.27 (Condyloma in 2007 in female vs. CC incidence rate in 2015). In regard to Gonorrhea incidence rate, correlation coefficients ranged from −0.14 (Gonorrhea in 2010 in female vs. CC incidence rate in 2017) to 0.43 (Gonorrhea in 2000 in female vs. CC incidence rate in 2015). Between the incidence rates of Herpes and those of CC, the correlation coefficients ranged between −0.26 (Herpes in 2010 in female vs. CC incidence rate in 2017) to 0.37 (Herpes in 1997 vs. CC incidence rate in 2016). 

Between medical examination rates and the CC incidence rate, negative correlations were coherently observed regarding the screening test rate, ranging from −0.30 (screening test rate in 2013 vs. CC in 2013) to −0.02 (screening test rate in 2016 vs. CC in 2015). On the other hand, positive and negative correlations were observed regarding the detailed examination rate, ranging from −0.21 (detailed examination rate in 2017 vs. CC in 2015) to 0.08 (detailed examination rate in 2014 vs. CC in 2013).

Between socio-demographic factors and the CC incidence rates, negative correlations were coherently observed regarding disposable income (−0.28 in CC 2015 to −0.05 in CC 2017), average economic surplus (−0.44 in CC 2016 to −0.16 in CC 2013), and smoking rate (ranging from −0.06 in CC 2016 to −0.16 in CC 2015). Proportion equal to or under high school graduate (−0.09 with CC in 2013 to 0.03 with CC in 2016) showed either positive correlations or negative correlations with the incidence rates of CC. We did not include the proportion equal to or under high school graduate as an explanatory variable for GLM due to the weakness in the correlation with CC incidences (|*r*| < 0.10).

### 3.2. Generalized Linear Modeling and the Estimation of the Effect of Screening

We observed the overdispersions in all the fitting processes of Poisson distribution and adopted negative binomial distribution for their error distributions. From the total 1088 constructed models, the lowest AICs in each model setting classified by the use of STD data and the time-lag between the STD incidences and the CC incidence are shown in Table 2. From these, the lowest AIC (2146.5) was obtained in the model with the “single-year STD data of both male and female” setting, with “15-year time-lag between the STD incidences and the CC incidences”.

**Figure 2:**
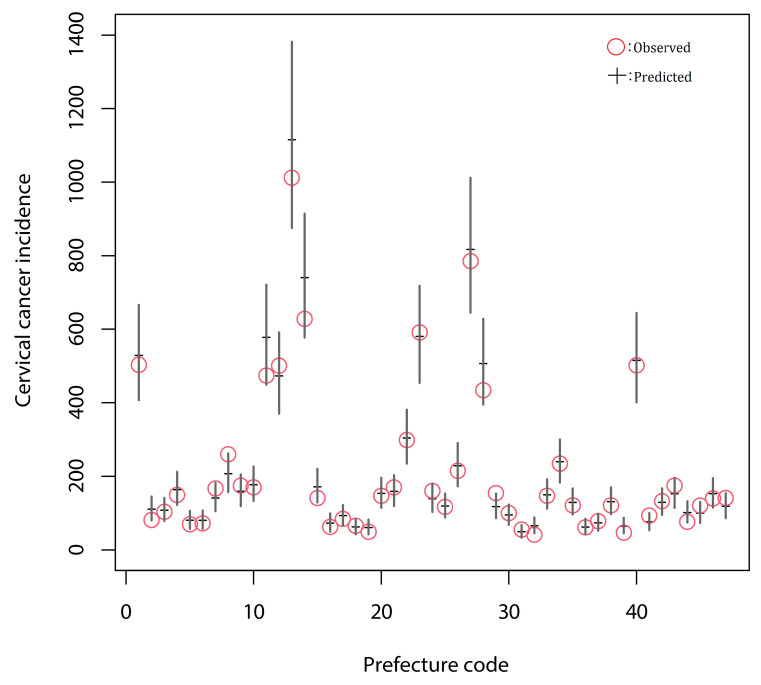
The predicted and observed incidences of cervical cancer in 2013 in 47 prefectures of Japan. The red circle denotes the observed CC incidence in each prefecture. The black horizontal bar and vertical solid line demonstrate the estimated CC incidence and its 95% confidence intervals. The relationships between observed and estimated prefectural CC incidences in other years are not shown here.

The explanatory variables and the regression coefficients in the model with the lowest AIC are shown in Table 3. STDs that were selected as explanatory variables (with 15-year time-lag with the CC incidences) and their regression coefficients were as follows: Chlamydia in female (0.0028, 95%CI: 0.0011–0.0044), Condyloma in female (−0.0128, 95%CI: −0.0260–0.0003), Gonorrhea in male (0.0061, 95%CI: 0.0039–0.0084), and Condyloma in male (−0.0207, 95%CI: −0.0330–−0.0084). This means the incidence rate of Chlamydia in female and that of Gonorrhea in male were associated with the increase of the incidence of CC with a 15-year time-lag, and Condyloma in both female and male may have contributed to decreasing the incidence of CC with a 15-year time-lag. As for other possible socio-demographic risk factors, the average economic surplus and the smoking rate were selected as explanatory variables, and their regression coefficients were estimated to be −0.0025 (95%CI: −0.0034–−0.0016) and −0.0043 (95%CI: −0.0052–−0.0034), respectively. Among the factors related to the medical examination, only the screening test rate was selected, showing a negative contribution to the incidences of CC. The regression coefficients of screening test rate were estimated to be −0.0101 (95%CI: −0.0150–−0.0053). We have confirmed the fitting of the lowest AIC model to the observed prefectural-level incidences of CC (Figure 2). The correlation coefficient between the estimated and observed incidence was 0.992.

**Figure 3:**
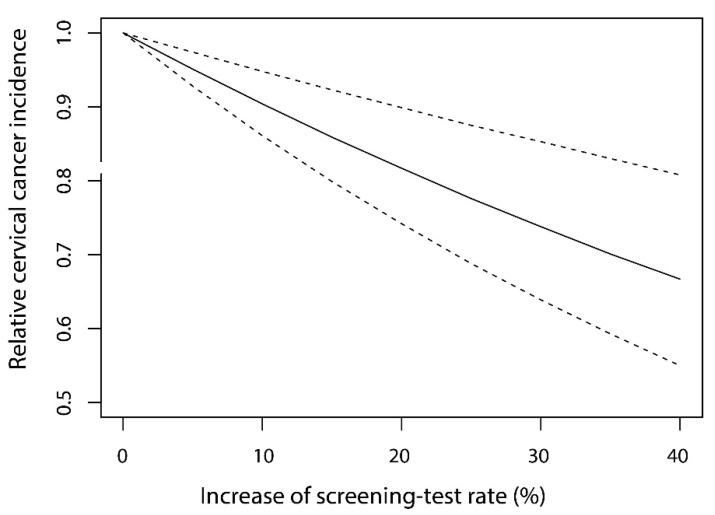
The prediction of decrease in the relative cervical cancer incidence by the increase of screening test rate.

Using the estimate of the regression coefficient in the lowest AIC model, we also predicted the relative change in CC incidence influenced by the increase of the screening test rate (Figure 3). As the screening test rate increased by 5%, 10%, 20%, and 40%, the decreasing rates of CC incidences were predicted to be 4.9% (95%CI: 2.6–7.2%), 9.6% (95%CI: 5.2–13.9%), 18.3% (95%CI: 10.1–25.8%), and 33.3% (95%CI: 19.2–45.0%), respectively.

## 4. Discussion

In the present study, we explored the associations between the incidence of CC in Japan and the incidence rates of four types of STDs (i.e., Chlamydia, Herpes, Condyloma, and Gonorrhea) in both sexes, medical examination rates (i.e., screening test rate and detailed examination rate), and socio-demographic factors (i.e., smoking rate, proportion equal to or under high school graduate education level, disposable and average economic surplus) as possible risk factors, taking into account the chronological relationship between the development of CC and the exposure to those possible risk factors. From the correlation analyses, screening test rate, economic status (disposable income and average economic surplus), and smoking rate showed coherent negative correlations with CC incidence, while all STD incidence rates, smoking rate, detailed examination rate, and proportion equal to or under high school graduate showed either negative or positive correlations depending on the chronological conditions. The contributions of possible risk factors for the prediction of the incidence of CC were also modeled by constructing negative binomial GLMs. Considering the 15-year time-lag between exposure to STDs and the onset of CC, the model clarified the positive contribution of Chlamydia in female and Gonorrhea in male, and the negative contribution of Condyloma in male and female, average economic surplus, smoking rate, and screening test rate for the prediction of CC incidence rate. To our limited knowledge, the present study is the first study that has (i) analyzed the association between population-level CC incidence and the incidence rate of STDs including data from both sexes, (ii) analyzed the chronological relationships between the incidences of STDs and those of CC, and (iii) quantitatively estimated the effect of medical examination on decreasing the incidence of CC.

The compatibility of positive correlation and negative correlation between the incidence of each STD and that of CC demonstrated the complex relationships between the STD infections and the development of CC. The direction of association varied depending on (i) the type of STD and (ii) the time difference between incidence of STD and that of CC. As for (i), the number of positive correlations with r>0.10 and that of negative correlations with r<−0.10 were 171 and 14 in Chlamydia, 35 and 57 in Condyloma, 207 and 4 in Gonorrhea, and 173 and 8 in Herpes (Supplementary Table S1). The incidence rates of Condyloma showed a higher frequency of negative correlation with incidence rate of CC compared to other STDs. Regarding (ii), incidence rates between 1992 and 1999 in Chlamydia in females tended to show a negative correlation, however, after 1999, the positive correlations increased. We could not identify the reason this phenomenon was observed. 

In the result of negative binomial GLMs, the positive contribution of Chlamydia in females and Gonorrhea in males was observed. The positive associations between the incidence of STDs and that of CC have often been reported in previous studies [47,48,49,50,51,52] as associations between HPV and other STDs, globally. In particular, Chlamydia and Herpes-simplex infections have often been listed as risk factors for HPV infection in a prospective cohort study [43] and a retrospective cross-sectional study [47]. Compared to these two STDs, the evidence of the possible association between Gonorrhea and the incidence of CC was less frequently reported; to our knowledge, only a cross-sectional study in the USA reported a high odds ratio of HPV infection in the female population who had experienced Gonorrhea infection compared to those who had not, suggesting the co-infection of Gonorrhea and HPV was considered to be a possible risk factor of subsequent development of CC [53]. Bearing in mind that the present study was based on the population-level ecological study using surveillance data, our findings may add evidence for the possible positive association between the infection of Gonorrhea and the development of CC. The result that Gonorrhea was selected as the predictor of the GLM while Herpes in either sex was not selected in the present study was intuitively interpretable. The surveillance data of Herpes may include recurrent infection and not clearly reflect the risky sexual behavior as the major route of HPV infection. To predict the risk of the development of CC, the surveillance data of Gonorrhea may possibly be useful with the data of Chlamydia infections. This may also imply that improving people’s awareness to prevent the infection of the above-mentioned STDs (i.e., avoiding risky sexual behavior) may help to reduce the incidence rate of CC.

In contrast, the negative contribution of Condyloma to CC incidence rate was observed in our model. Some previous studies provide an explanation for this observation: possible interference between the HPV infection related to Condyloma (i.e., LR-HPV; HPV 6 and 11) and HRHPV related to the development of CC (HPV 16 and 18) have been reported [22,54,55,56]. The result in the present study suggests that past exposure to the LR-HPV may also decrease the incidence of CC in Japan.

Concerning the medical examination, we confirmed that the increase in screening rate may contribute negatively to the increase of CC incidence, and the effect is expected to be an almost one-to-one relation, especially in the lower percentage of increase in the screening test rate (shown in Figure 3). This result implies that most potential patients (i.e., pre-cancerous patients) in the population newly tested by the screening test can be detected before the development of cervical cancer, which shows the high effectiveness of the screening test. The result was consistent with the high effectiveness of screening for the detection of precancerous patients in populations reported from case-control studies in the USA [57,58], the UK [59], and Australia [60], and observational studies in other countries [61]. We consider that our study added ecological study-based evidence for the high effectiveness of the screening test.

On the other hand, the limitation in the strategy that fully relies on the screening test should be considered. For instance, in the lack of acceptance to HPV vaccination, MHLW in Japan have tried to increase the screening test rate for the prevention of CC [62]. They recommended taking the screening test to women over 20 years old, aiming to achieve a 50% or more screening rate based on the “National Basic Plan to Promote Cancer Control Program in Japan (Plan 3)” [63]. Considering that the current screening test rate is distributed between 27% and 47% among prefectures [37], the aim will be achieved when the screening rate increases by approximately 5–25%. In our estimation, however, such increases may provide only a 4.9–22.4% relative reduction in CC incidence and result in a limited effect. When considering the difficulty in increasing the screening rate in the limited resources for cancer screening in Japan [64], the increase of screening rate alone may not play a crucial role for the decrease of CC incidence compared to the known effect of HPV vaccination that can drastically decrease the CC incidence in many countries [65,66,67]. The combination of the increase of screening rate and that of vaccine coverage will be important for more effective prevention of CC in any country.

As for other variables that were selected in the GLM, negative contributions to the increase of CC incidence by average economic surplus and smoking rate were observed. The former is consistent with the reported results in previous studies that show that people with a higher economic status showed lower risk of development of CC [68,69]. Similar results have been widely reported from population studies in low-income group countries [51], but our results revealed that the same phenomenon exists in Japan, a high-income group country. Although there may be possible confounding factors, such as the awareness of HPV risk being lower in prefectures with lower economic status, governments in those prefectures need to put further effort into the prevention of CC.

The negative association between the smoking rate and CC incidence in the correlation analysis and the negative contribution of smoking rate to the CC incidence in the regression analysis observed in the present study do not concur with the widely accepted concept of “increasing the risk of CC development by smoking.” The concept was supported by scientific evidence provided from many studies, not only by epidemiological studies including the research that targeted Japanese women [70,71], but also by experimental studies [72]. We could not find any possible explanation for the inconsistency between the result in the present study and those in previous research. Hence, a further study is needed to tackle this inconsistency and clarify the background mechanism for the observation in the present study.

In addition to the difficulty in the interpretation of the relationship between smoking rate and CC incidence, there are some other limitations in the present study. First, due to a change in the government cancer registration system, the incidence data of CC from 2013 to 2015 were collected based on the Regional Cancer Registry System, and those from 2016 to 2017 were collected based on the Cancer Registry and Statistics system. Although we confirmed that the trend in the distribution of CC incidence among prefectures was not significantly different between 2013–2015 and 2016–2017 (*p* = 0.26, χ^2^ test), the change may possibly bias the data. Second, because the age-specific CC incidence was not available at the prefecture level, we could not include the difference of age structure of prefectural populations in our analyses. The difference in age structure may bias the difference in CC incidences between the prefectures, and hence, not only the region-specific but also age-specific data are required for future analyses. Third, in the prediction of CC incidence, we extracted the STD data only in the same year(s). This is because (i) we primarily expected that the incidence of STDs represented the exposure to HPV through sexual behavior in females rather than the maintenance of HPV, and (ii) to avoid the loss of identifiability and a high-computational burden in the estimation. If there is a heterogeneity in the influence of STDs to the persistence of HPV in cervical environments, we did not clearly include that in our analyses. For further clarification, we expect that the mechanism of CC development will be elucidated from the viewpoint of the interaction (i.e., co-infections and interferences) among multiple STD pathogens. Fourth, there could be a long interval between the exposure to HPV and the diagnosis of cervical cancer, which enabled women to move across prefectures. Since the overall data we used in Japan did not track the situation of every patient, this led to a limitation. Fifth, we used the Random Forest algorithm for the imputation of missing data. Due to the high computational burden in the present study, we could not conduct the sensitivity analysis for the imputation. Although the percentage of missing data in the present study (1.81%) was not large compared to the rule of thumb for the “ignorable percentage” (e.g., 5% [73]), the variance of imputation should be taken into account in further studies.

## 5. Conclusions

Our study revealed that the increase of the screening rate can help decrease CC incidence, and the effect for the prevention of CC development seems to be high in the population that took the screening test. On the other hand, the effect of CC prevention relying only on the screening test was considered to be limited. As reported in countries with high HPV vaccine coverage, not relying solely on the screening test, the combination of increasing the screening rate and the coverage of the HPV vaccine will be a more efficient countermeasure to drastically decrease CC incidence.

## Figures and Tables

**Table 1:** Variables used in analysis with their source and period.

Data	Period	Source
Incidence of CC	2013–2017	Cancer Registry and Statistics [33,34]
Incidence of STDs Condyloma, Chlamydia, Gonorrhea, Herpes	1993–2012	National Epidemiological Surveillance of Infectious Diseases [35,36]
Screening test rate for cervix	2013, 2016	Comprehensive Survey of Living Conditions [37]
Detailed examination rate for cervical diseases	2013, 2016	Comprehensive Survey of Living Conditions [37]
Smoking rate	2001, 2004, 2007, 2010, 2013	Comprehensive Survey of Living Conditions [37]
Disposable income	2015	Statistics Bureau of Japan [38]
Average economic surplus	2014	Ministry of Land, Infrastructure, Transport and Tourism [39]
Proportion whose educational level is equal to or under high school graduate (education level)	2012	Population Census [40]
Equal to or over 25 years old female population in each prefecture	2015	Statistics Bureau of Japan [41]

**Table 2:** The different settings in the use of STD data and the time-lag between the STD incidences and CC incidences with the Akaike information criterion calculation.

Years of STD Incidences that Were Averaged *		Type of STD Data			
		All STDs	Female STDs Only	Male STDs Only	STDs in Both Genders
1 **	Time-lag	14	14	15	15
	AIC	2155.1	2177.3	2162.9	2146.5 ***
3	Time-lag	16	15	16	16
	AIC	2150.8	2174.5	2160.8	2151.0
5	Time-lag	16	16	18	16
	AIC	2150.0	2171.5	2160.9	2150.7
7	Time-lag	18	17	19	17
	AIC	2150.6	2170.0	2162.2	2149.9
9	Time-lag	19	19	20	19
	AIC	2151.5	2168.6	2163.8	2149.6
11	Time-lag	17	20	20	20
	AIC	2153.1	2168.9	2166.7	2151.1
13	Time-lag	18	20	20	18
	AIC	2153.1	2169.4	2168.8	2152.4
15	Time-lag	19	20	19	19
	AIC	2154.1	2169.9	2168.8	2152.5

* We also calculated the 2, 4, 6, 8, 10, and 12 years, however, did not describe the results here because of the limitation of space. ** Single year (i.e., no moving average). *** The lowest AIC among all the models.

**Table 3:** The explanatory variables and the estimates of regression coefficients in the model with the lowest AIC among the 1088 negative binomial regression models for cervical cancer incidence.

Variables	Regression Coefficient	95%CI	*p*
Intercept	−7.0399	−7.3886–−6.6912	<0.001
Chlamydia (female)	0.0028	0.0011–0.0044	<0.001
Condyloma (female)	−0.0128	−0.0260–0.0003	0.056
Condyloma (male)	−0.0207	−0.0330–−0.0084	0.001
Gonorrhea (male)	0.0061	0.0039–0.0084	<0.001
Average economic surplus	−0.0025	−0.0034–−0.0016	<0.001
Smoking rate	−0.0209	−0.0301–−0.0116	<0.001
Screening test rate	−0.0101	−0.0150–−0.0053	<0.001

## Data Availability

The data presented in this study are openly available in Cancer Registry and Statistics (https://ganjoho.jp/reg_stat/statistics/data/dl/index.html accessed on 20 October 2020; in Japanese), National Epidemiological Surveillance of Infectious Diseases (https://www.niid.go.jp/niid/en/ accessed on 20 October 2020), Comprehensive Survey of Living Conditions (https://www.e-stat.go.jp/stat-search/files?page=1&toukei=00450061 accessed on 20 October 2020), and national Population Census (https://www.stat.go.jp/english/data/kokusei/index.html accessed on 20 October 2020), Average economic surplus calculated by Ministry of Land, Infrastructure, Transport, and Tourism (https://www.mlit.go.jp/policy/shingikai/content/001389727.pdf accessed on 20 October 2020) (in Japanese).

## Supplementary Materials

The following are available online at https://www.mdpi.com/article/10.3390/cancers13194784/s1, Table S1: One hundred and thirty-six chronological patterns of STD data and CC data. Table S2: Correlation matrix among cervical cancer incidence and possible risk factors.
